# The dollars and sense of economic incentives to modify HIV-related behaviours

**DOI:** 10.7448/IAS.18.1.20724

**Published:** 2015-10-17

**Authors:** Andrew R Zullo, Katherine Caine, Omar Galárraga

**Affiliations:** Department of Health Services, Policy, and Practice, School of Public Health, Brown University Providence, RI, USA

## Introduction

In 2012 alone, there were 2.3 million new HIV infections globally, of which 1.9 million were in countries marked by poverty [1]. Even in the affluent United States, approximately 56,000 individuals have been newly infected each year since 2006, with vulnerable groups like sexual and ethnic minorities disproportionately at risk [2]. The burden of HIV/AIDS in disadvantaged populations underscores the structural and economic factors that may serve as intervention targets for changing behaviour to prevent or treat HIV. Literature has suggested that both affluence and poverty can be associated with increased risk of HIV infection, but there are documented, vulnerable subsets of the population for whom poverty induces more HIV risk behaviours [3–5]. The field of behavioural economics provides a theoretical framework to understand (1) the conditions under which risky decisions are amenable to intervention and (2) how to capitalize on potential intervention targets [6, 7].

## Economic incentives

In the past decade, economic incentives (EIs) have emerged as a feasible and potentially cost-effective structural intervention from behavioural economics [8, 9]. Commonly, EIs use a financial reward to incentivize desirable behaviours that promote improved health outcomes. Common incentivized behaviours include returning to the HIV clinic and adhering to an antiretroviral therapy regimen. EIs come in two forms: conditional – the recipient receives the incentive only if he/she achieves predefined endpoints – and unconditional – he/she receives it regardless [10]. The exact mechanism for how EIs impact health is poorly understood, but research suggests that additional financial resources from EIs may improve material conditions, enhance social capital and reduce or remove constraints on choice, cognition and opportunity to instil agency in individuals’ lives [11–13].

Studies show that EI interventions do not need to supply large rewards to reap benefits; often for those with low socio-economic status, a small sum represents a large proportion of their income [10, 14–23]. Prior studies suggest that incentive design (e.g. lottery, conditional on school attendance), recipient (e.g. female vs. male head of household) and, perhaps most importantly, the relative poverty of the recipients all may modify the effect of EIs on HIV-related outcomes [10, 14–23]. The structure of the EI programme matters, especially since EIs have actually increased HIV vulnerability in circumstances where the incentive could be used in a harmful way, such as to purchase riskier sex [24].

## Selected recent examples

In 2008, Thornton evaluated an experiment in rural Malawi in which adults were randomly assigned to receive a voucher worth one day's wages if they returned to a clinic to obtain HIV test results [21]. Individuals in the incentive group were twice as likely to return to the clinic. Another 2011 study of a conditional incentive ($0, $4 or $16 voucher) to remain HIV negative in Malawi produced the following: (1) an increase in sexual risk behaviour among men one week after receiving the incentive and (2) no effects on risky sexual behaviour at one year of follow-up [24]. In 2012, de Walque *et al*. assessed a cash transfer programme [high ($20) vs. low ($10) vs. no incentive] among adults in Tanzania wherein payment was conditional on negative sexually transmitted infection (STI) results [18]. The high incentive group showed a significant reduction in STI prevalence, but the low incentive group had no measurable reduction; overall, the study was unpowered to assess any effect on HIV incidence. Also in 2012, Baird *et al*. assessed the effect of cash transfers (both unconditional and conditional on school attendance) on HIV prevention among adolescent girls in Malawi [19]. They found a decreased prevalence of HIV in the incentive groups after 18 months with no difference by incentive type. A 2014 study by Thirmurthy *et al*. assessed a one-time food voucher incentive for men to undergo circumcision in Kenya, which reduces HIV incidence up to 60% [25]. They documented modest increases in circumcision uptake after two months. Lastly, in 2015, Nyqvist *et al*. showed that a lottery programme in Lesotho that was conditional on having negative test results produced a 21.4% reduction in two-year HIV incidence among adults [22]. For future studies, targeting the interventions to the poorest sub-population at highest risk of HIV infection, such as sex workers and other vulnerable groups, is one potential strategy to mitigate the inadequate statistical power that affects some EI studies.

## EIs as government policy

Governmental policy could be a platform to scale up EIs and have a lasting, global effect. An effective policy could target vulnerable populations in the HIV epidemic in order to reduce their poverty burden. These populations are traditionally overlooked and have some of the highest prevalences of HIV: men who have sex with men (MSM), adolescents, injection drug users and sex workers. In Mexico City, for example, researchers distributed surveys grounded in behavioural economics to better identify the monetary threshold for an effective stipend among high-risk MSM [15, 16, 26]. Using the results, a government body could provide an incentive to eligible individuals or families, conditional on specific outcomes. Such a longitudinal intervention could encourage healthier behaviours and give participants the freedom to address economic insecurity in the way it most influences their lives – improving educational opportunities, paying back loans, utilizing public transportation. In these cases, scale plays an integral role: governments alone may have the capacity to implement and monitor such programmes. However, the difficulty of implementing such programmes for populations that are often criminalized and marginalized by the governments of many countries cannot be understated: many individuals will not disclose their membership in a vulnerable population and cannot be identified for inclusion in a programme. For programmes that are successfully implemented, the political economy of the government rather than empirical evidence can determine the structure of the programme (i.e. conditional vs. unconditional incentive) [27].

## Summary

We need scalable, evidence-based programmes to prevent HIV and increase healthy behaviours in vulnerable populations characterized by poverty. Behavioural economic incentive programmes are a viable option and may already be available for incorporation into government policy [16, 28]. Future research should focus on how to best structure and successfully implement these programmes to maximize effectiveness and address political challenges.
